# LC3-Associated Phagocytosis (LAP): Connections with Host Autophagy

**DOI:** 10.3390/cells1030396

**Published:** 2012-07-30

**Authors:** Shu-chin Lai, Rodney J. Devenish

**Affiliations:** 1 Department of Biochemistry and Molecular Biology, Monash University, Clayton campus, Melbourne, Victoria 3000, Australia; Email: Shu-chin.Lai@monash.edu; 2 ARC Centre of Excellence in Structural and Functional Microbial Genomics, Monash University, Clayton campus, Melbourne, Victoria 3000, Australia

**Keywords:** autophagosome, autophagy, LC3, phagocytosis, LC3-associated phagocytosis (LAP)

## Abstract

Autophagy is an intracellular degradative process with a number of roles, one of which can be the protection of eukaryotic cells from invading microbes. Microtubule-associated protein light-chain 3 (LC3) is a key autophagy-related protein that is recruited to the double-membrane autophagosome responsible for sequestering material intended for delivery to lysosomes. GFP-LC3 is widely used as a marker of autophagosome formation as denoted by the formation of green puncta when viewed by fluorescence microscopy. Recently, it has been demonstrated that LC3 can be recruited to other membranes including single-membrane phagosomes, in a process termed LC3-associated phagocytosis (LAP). Thus, the observation of green puncta in cells can no longer, by itself, be taken as evidence of autophagy. This review will clarify those features of LAP which serve to distinguish it from autophagy and that make connections with host autophagic responses in terms of infection by microbial pathogens. More specifically, it will refer to concurrent studies of the mechanism by which LAP is triggered in comparison to autophagy.

## 1. Introduction

The phagocytic activity of professional phagocytes, such as macrophages and neutrophils, plays an essential role in innate immunity [1]. The engulfment of a microbial pathogen by a phagocyte results in formation of an intracellular vacuole, termed the phagosome. The phagosome containing the microbe eventually fuses with lysosomes, the lumen of which provides a range of acid hydrolase enzymes that collectively digest the content of the phagosome [2,3]. Autophagy is a multi-step process by which proteins and other molecules are sequestered in double-membrane vesicles, autophagosomes, which ultimately fuse with lysosomes to facilitate degradation and thereby regulate cellular homeostasis. The cargo degraded in autophagosomes can include older of defective organelles. There are at least three types of autophagy: macroautophagy, microautophagy and chaperone-mediated autophagy [4]. This review will focus on macroautophagy (hereafter autophagy) since that is the process used by cells to degrade organelles or large internalized particles.

**Figure 1:**
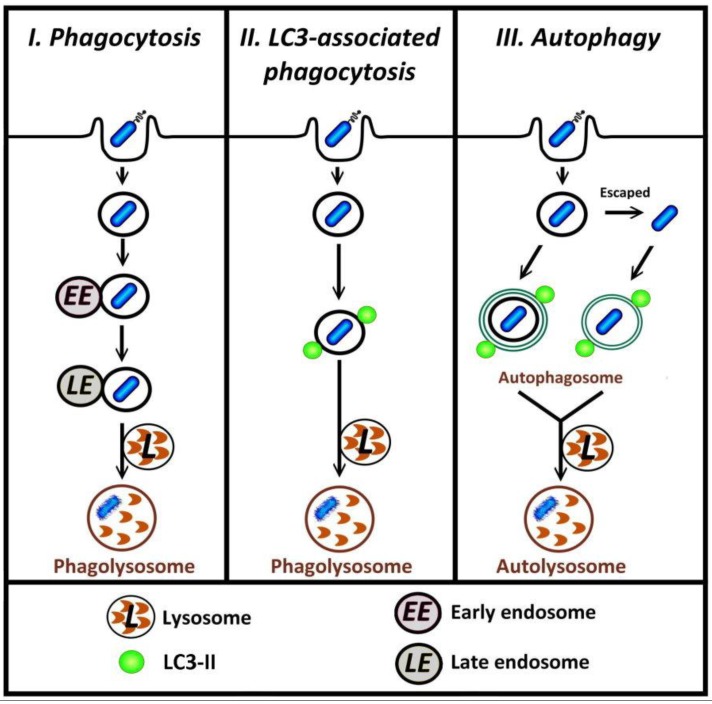
Different pathways by which bacteria in phagosomes can be degraded. I, in typical phagocytosis of bacteria, the phagosome may undergo fusion with endosome and lysosome (phagosome maturation) such that the bacterium is contained within a phagolysosome, the degradative compartment. II, in LC3-associated phagocytosis, autophagy proteins including LC3-II are recruited to the surface of phagosomal membranes; these vesicles subsequently fuse with lysosome. III, bacteria that are retained in, or escape from, phagosome can be targeted by autophagy.

Several recent reports describe investigations into the relationship between autophagy and infection with a particular focus on the mechanisms for the recognition of invading pathogens by host autophagy [5,6,7]. The autophagic machinery following the entry of the pathogen into cells can target either bacteria-containing phagosomes, or free cytosolic bacteria [8,9,10,11]. In both phagocytosis and autophagy, the recruitment of proteins to membranes is a key process that can lead to the subsequent degradation of intracellular pathogens (Figure 1, paths I and III). Recent research has shown that microtubule-associated protein light-chain (LC3), previously considered to be exclusively an autophagic marker, is also involved in LC3-associated phagocytosis (LAP) [12,13,14,15,16,17] (Figure 1, path II). In LAP, distinct from the conventional autophagic pathway where LC3 is incorporated into the double-membrane autophagosome, LC3 is recruited directly to the single-membrane phagosome [12,16,18]. LC3 can also associate with other cellular components including microtubules [19] and the osteoclast ruffled border observed in bone resorption [20]. This review first will provide a brief overview of the autophagy pathway, including the role of LC3 in autophagosome formation, followed by details of LC3-associated phagocytosis. At the end of this review, future research directions regarding the potential mechanism for triggering LAP will be discussed.

## 2. Autophagy

The mechanism of autophagy is comprised of several steps and at least 30 autophagic-related (Atg) proteins. The cellular components or invading pathogen to be degraded are ‘captured’ by a membrane structure that is initiated in response to signaling events (the phagophore) and then extended such that the completed membrane encloses the targeted material in a double-membrane autophagosome [21,22,23]. The formation of the phagophore requires the activity of the ULK-Atg13-FIP200 complex, while its elongation requires the class III phosphatidylinositol 3-kinase (PI3K) complex consisting of Vps34, Beclin 1 and Vps15. Two ubiquitin-like conjugation systems also contribute to autophagosome formation [23,24,25]. One of these systems requires the ubiquitin-like protein LC3 and is considered essential for autophagy. The cytosolic form of LC3 (designated LC3-I) is conjugated to phosphatidylethanolamine (PE) and in this form (designated LC3-II) it is recruited to the growing autophagosome membrane [26]. Prior to the fusion of autophagosomes with lysosomes, the LC3-II located on the outer autophagosomal membrane is released by deconjugation of PE from LC3 by the proteolytic activity of Atg4. By contrast, LC3-II associated with the inner membrane is retained, although eventually degraded by the acid hydrolases within the autophagolysosome [27]. 

Interestingly, mammalian cells contain at least 8 LC3 homologues divided into two subfamilies based on amino acid sequence. The first LC3 subfamily comprises LC3A (including 2 splicing variants), LC3B and LC3C [28]. The second, the GABARAP/GATE subfamily, comprises GABARAP, GABARAPL1, GATE-16 (aka GABARAPL2) and GABARAP-L3 [29,30]. LC3B is the protein that is widely used as a fusion with GFP (GFP-LC3) to follow autophagosome formation using fluorescence microscopy [31]. Aside from LC3B the other family members have not been studied in any detail and it has been unclear whether they each have a distinct and crucial role in autophagy. Recent work has indicated that the two subfamilies act differently at early stages of autophagosome biogenesis. Thus, the LC3 subfamily is required for elongation of the phagophore membrane, whereas the GABARAP/GATE subfamily is required for a later stage in autophagosome maturation [32]

## 3. LC3-Associated Phagocytosis

**Figure 2:**
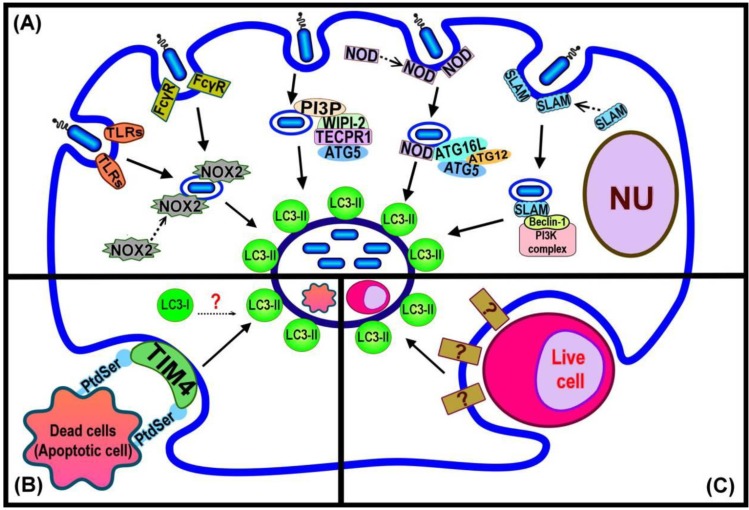
Summary of LC3-associated phagocytosis (LAP) pathways and current evidence regarding the events that trigger LC3 recruitment. (**A**) LAP triggered by bacterial infection depends upon different host cell surface markers, or cytosolic pathogen sensing signals acting either by direct induction, or binding to autophagy component proteins; (**B**) LAP function in dead cell clearance requires the PS receptor TIM4 for induction of LC3 recruitment to the phagosome; (**C**) LAP for cell-eats-cell, entosis, is induced by an unknown mechanism.

LC3 recruitment to the phagosome membrane has been shown to occur in the phagocytosis of pathogens, apoptotic bodies or entotic bodies (arising from live cell phagocytosis), see Figure 2. The first report of such recruitment showed that the engulfment of *Escherichia coli* and yeast by RAW macrophage-like cells expressing GFP-LC3 induced the translocation of LC3 to phagosomes within 5 to 10 minutes of internalization of microorganisms [18]. Similarly, infection of either phagocytic neutrophils or non-phagocytic mouse embryonic fibroblast (MEF) cells, each expressing GFP-LC3, by *Salmonella typhimurium* also resulted in the recruitment of GFP-LC3 to bacteria-containing phagosomes [14,33]. Furthermore, by following the indirect immunofluorescence staining of endogenous LC3, Lerena and Colombo [15] showed that LC3 decorated *Mycobacterium marinum*-containing phagosomes after infection of RAW cells. In the case of *Burkholderia pseudomallei* infection of GFP-LC3 expressing RAW macrophage cells, the intracellular bacteria reside within LC3-positive phagosomes [13], from which they later escape into the cytosol. Intriguingly, the escaped *B. pseudomallei* are not subject to autophagy. 

In these instances of pathogen-related LAP, bacterial viability is required in order to initiate the recruitment of LC3 to the phagosome [15]. However, it is evident that in other cases LAP does not require the viability of the internalised particles, such as corpses from cells having undergone programmed cell death. The phagocytosis of dead cells arising from the activity of three different programmed cell death pathways, apoptosis, necrosis and RIPK3-dependent necrosis, were shown to give rise to the recruitment of LC3 to phagosomes [16]. Similarly, in *Caenorhabditis elegans*, LGG-1 (homolog of LC3) is recruited to the apoptotic Q neuroblast (Q cell) corpse [34]. 

Entosis is a live-cell engulfment pathway occurring between two neighbouring cells, one of which will be actively engulfed by the other to form a ‘cell-in-cell’ structure [35]. The internalised cell in a phagosome is shown to be decorated by LC3 and itself undergoes programmed cell death [12]. The same report also demonstrated that micropinosomes, and endocytic vesicles filled with extracellular fluid, also recruited LC3 [12]. Hence, LAP has a number of different roles in the cell that include maintenance of cellular homeostasis and protection against invading pathogens. 

## 4. Initiating LAP

The exact mechanism(s) responsible for triggering LAP is still to be determined. A summary of current knowledge is presented in Figure 2. Invading pathogens are recognized by mammalian cells through receptors found either at the cell surface (eg, trans-membrane Toll-like Receptors; TLRs), or in the cytoplasm (Nod-like Receptors (NLRs) [36,37]. The recognition of bacterial LPS, by host TLR2 and 4, initiates the engulfment of the invading pathogen by the plasma membrane to form the phagosome [38]. The newly formed phagosome is rapidly decorated by LC3 [18] (Figure 2A). Proteomics analysis of purified phagosome membranes in cells that have taken up differing cargoes by phagocytosis has revealed that the absence of TLR signaling leads to failure of LC3 recruitment to phagosome membranes [18,39]. Moreover, Huang *et al*. [14] showed that the activation of NOX2 NADPH oxidase by TLRs or Fcγ receptors (FcγR) in order to produce microbiocidal reactive oxygen species (ROS) resulted in the recruitment of LC3 to the phagosome. Both TLR- or FcγR-dependent NOX2 activity triggered the formation of LC3-associated phagosomes that eventually fused with lysosomes and the invading pathogens were degraded. Although little is known concerning the direct links between pathogen-related factors and LAP, the available evidence demonstrates the recruitment of some autophagy proteins, such as Beclin 1, that normally act upstream of the ubiquitin-like conjugation pathway that generates LC3-II to pathogen-containing phagosomes. Members of the SLAM (signalling lymphocyte-activation molecule) family are involved in the regulation of phagosome maturation and NOX2 activity by interacting with the Vps34-Beclin 1-Vps15 complex [40], as a possible means of subsequently recruiting LC3 to the phagosome. The NLRs are a family of cytosolic proteins that recognize intracellular pathogens, through the peptidoglycans on the surface of the bacteria, and initiate the proinflammatory response [41]. NOD1 and NOD2 recruit Atg16L to the site of bacterial invasion at the plasma membrane, potentially facilitating the recruitment of LC3-II to the phagosome membrane [42]. Tectonin domain-containing protein, Tecpr1, has been demonstrated to trigger selective autophagy. Tecpr1 targets bacteria via directly binding Atg5 and then with the co-operation of WIPI-2, a phosphatidylinositol 3-phosphate (PI3P) binding protein homologous to yeast Atg18, recruits LC3 to the phagophore [43]. PI3P is generated from the phosphorylation of phosphatidylinositol (PI) via Vps34, which is present on both phagophore [44] and phagosome membranes [3]. Thus, a model that requires the sequential interaction of PI3P, WIPI-2, Tecpr1 and Atg5 has been purposed to enhance the autophagic targeting of bacteria, protein aggregates and damaged mitochondria [45]. It is presently unknown whether Tecpr1 also triggers LC3 recruitment to the phagosome although it is known that this does require the activity of PI3K [18]. 

The clearance of dead cells by LAP (Figure 2B) requires the phosphatidylserine (PS) receptor T cell immunoglobulin domain and mucin domain protein-4 (TIM4). Primary macrophages with reduced expression of TIM4 are deficient in dead cell-specific LAP. The actual signal from dead cells remains to be identified [16]. It is possible that the alteration of the membrane structure of the dying cell might itself play a role in triggering LAP. For example, PS, the major lipid component of the inner-leaflet of the plasma membrane, is known to become exposed on the cell surface during apoptosis and act as a signal to attract macrophages and induce phagocytosis [2,46]. Atg18 is recruited to the phagosome containing apoptotic Q cell corpses in *C. elegans* (in addition to LC3) [29], suggesting a linkage between PI3P binding at the membrane and recruitment of LC3 in this process [34]. In turn this would suggest similarity with events required for LC3 recruitment in the WIPI-2-Tecpr1-Atg5 dependent pathway of autophagy induction [45]. 

Ubiquitination of bacteria occurs as part of the host autophagic response to infection. Recent research indicates that several adaptor proteins, such as p62 and NDP52 serve to connect ubiquitin with LC3 facilitating the formation of the autophagosome membrane [47,48,49,50]. However, there is currently no evidence to suggest that initiation of LAP might rely on the ubiquitination of phagosome membrane components and the binding of adaptor proteins that subsequently bind LC3. An alternative mechanism for recruiting LC3 may relate to other recent observations concerning the mechanism of bone resorption in osteoclasts. It has been proposed that LC3 participates in the regulation of ruffled border (RB)-lysosome fusion process [51] in a Atg5, Atg7 and Atg4B-dependent manner, which facilitates recruitment of the lysosomal marker LAMP1 and endocytic marker Rab7 to the RB [20]. 

In autophagy, the mechanisms by which the membranes of preautophagosomal structures (PAS) are elongated and mature into fully formed autophagosomes are largely unknown. Recently, it was shown that the maturation of the early Atg16L1 precursors requires homotypic fusion, which is dependent on the soluble N-ethylmaleimide-sensitive factor attachment protein receptor (SNARE), vesicle-associated membrane protein 7 (VAMP7) together with partner SNAREs. This step regulates the size of the vesicles, which in turn appears to influence their subsequent maturation into LC3-positive autophagosomes [52]. In addition, clathrin plays a crucial role in endocytosis by which endocytic vesicles are formed and then transported within the cytosol [53]. It has also been demonstrated that the Atg16L1-positive precursor is associated with clathrin and that knockdown of clathrin inhibited autophagosome formation as observed by the decrease of LC3-II levels [54]. However, the presence of vesicles, capable of fusing with phagosomes or exchanging membrane with them to recruit LC3, has not been observed in LAP. Notably, in the study that led to the first report of LAP, the authors commented that it was possible the LC3-associated single-membrane structure might arise from the fusion of very small autophagosomes that first surround the phagosome and then fuse with the phagosomal membrane [18]. 

Some potential factors that are capable of triggering LAP are listed in Table 1. 

**Table 1:** Possible factors that trigger LC3-associated phagocytosis (LAP).

Bacterial ligands or cellular signal	Receptor	Link to autophagic-related pathway	Reference
Lipopolysaccharides (LPS)	TLRs (TLR2, TLR4)	Co-localization with LC3 on phagosomal membrane	[18]
Peptidoglycan	NLRs (NOD1, NOD2)	Direct binding with Atg16L	[42]
Reactive oxygen species (ROS)	TLR2, FcγR	Inhibition of NADPH oxidase decreased LC3 recruitment	[14]
Outer membrane proteins C and F of *E. coli*	SLAM	React with Vps34-Beclin 1-Vps15 complex; possibly direct binding with Beclin 1	[40]
Tecpr1	WIPI-2	An interaction between PI3P, WIPI-2, Tecpr1 and Atg5 followed by recruitment of LC3	[43]
Ubiquitin	Adaptor protein (P62, NDP52)	LIR and UBA motifs facilitate binding of ubiquitinated material to LC3	[47,48,49]
PS of dead cell membranes	TIM4	LC3 translocation triggered by phagocytosis of PS-containing liposomes in macrophages	[16]

## 5. Distinguishing LAP from Autophagy

**Figure 3:**
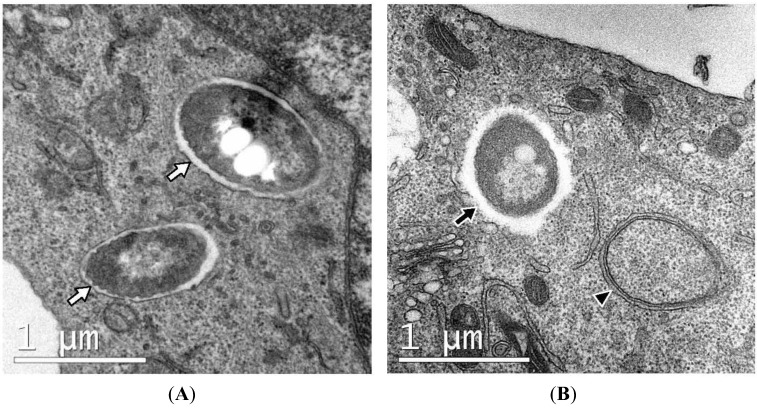
Ultrastructure of RAW macrophages expressing GFP-LC3 infected with *B. pseudomallei*. Intracellular bacteria are either sequestered within single-membrane phagosomes (white arrows in **panel A**) or free in the cytosol (black arrow, **panel B**). Single-membrane phagosomes can be clearly distinguished from double-membrane autophagosomes (arrowhead, **panel B**).

Currently, there is no readily applicable technique available to determine whether the presence of green puncta co-localized with bacteria in cells expressing GFP-LC3 represent LAP or canonical autophagy. The two methods available are not facile and require the ultrastructure observation of infected cells by TEM or tests to determine autophagy-related (Atg) gene dependency of co-localization of bacteria with green puncta. In regard to the use of TEM, the most significant ultrastructural difference that distinguishes LAP from canonical autophagy is that bacteria are enclosed within single-membrane phagosomes, instead of double-membrane autophagosomes (Figure 3) [13,15,16,18]. Single-membrane structures have been reported in non-phagocytic cells, although the mechanism of internalisation is considered to be endocytosis rather than phagocytosis [12,33]. A summary of published observations of single-membrane structures in bacterial infection that are indicative of LAP is presented in Table 2. 

The single known difference in requirement of known autophagy proteins between autophagy and LAP is that unc-51-like kinase 1 (ULK1) is not needed for LAP [16]. Although this difference provides a more convenient means for distinguishing between the two processes it still requires cell lines lacking expression of ULK1, or efficient knockdown of ULK1 expression. It is noteworthy that no exhaustive investigation of the requirement of Atg proteins for LAP has been published to date. It is possible that certain Atg proteins used in the initiation of canonical autophagy by nutrient starvation or pharmaceutical treatments may not be necessary for recruitment of LC3 to the phagosome or endosome, but this remains to be established. There is general agreement that LAP requires the Atg5-Atg12-Atg16L conjugating system during LC3 recruitment. Loss of any of the components of this system would be expected to abolish LAP function. Thus, knockout of either *ATG5* or *ATG7* significantly reduced the levels of LC3 surrounding the phagosomes containing invading bacteria [14,18,33], apoptotic bodies [16] or live cells [12]. Inhibition of PI3K by wortmannin or 3-methyladenine dramatically reduces LC3 recruitment to autophagosomes [55,56]; however it was evident that Atg5 and LC3 were still recruited to the *S. typhimurium*-containing phagosomes after treatment with wortmannin [33]. Rapamycin, a pharmaceutical inhibitor of mTOR and a well-documented inducer of autophagy, has been shown to induce the recruitment of LC3 to bacteria- or latex bead-containing phagosomes [15]. The results emerging from our studies regarding the infection of macrophage cells by *B. pseudomallei* have demonstrated an increased level of LAP after rapamycin treatment or starvation (another classic inducer of autophagy). However, in *BECLIN 1* knockdown cells the rapamycin response is Beclin 1-independent whereas the starvation response is Beclin 1-dependent [57]. 

**Table 2:** Evidence for the occurrence of LAP.

Phagocytic cells			
Phagocytosed Material	Cell Type	Morphological Features	Reference
Zymosan (dead yeast)	RAW264.7 murine macrophages expressing GFP-LC3	Cells are engulfed within single-membrane phagosomes even after starvation or treatment with rapamycin	[18]
Dead cells	CD11b^+^ F4/80^+^ primary macrophages derived from bone marrow of GFP-LC3+ mice	The vesicular structures containing apoptotic or necrotic cells are observed as being single-membraned by TEM observation at the time that the majority of dead cell-containing phagosomes co-localized with LC3-II	[16]
*Burkholderia pseudomallei*	RAW264.7-GFP-LC3	The LC3-positive structures that co-localize with bacteria are revealed as phagosomes by TEM.	[13]
*Mycobacterium marinum*	RAW264.7-GFP-LC3	Bacteria were within single-membrane components with the presence of typical autophagosomes nearby.	[15]
**Non-phagocytic cells**			
**Endocytosed Materials**	**Cell Type**	**Ultrastructure**	**Reference**
*Salmonella typhimurium*	MEF-GFP-LC3	Correlative light microscopy-electron microscopy (CLEM) observation of GFP-LC3 signals after infection displayed a single-membrane structure surrounding bacteria.	[33]
Entotic cells	MCF10A-GFP-LC3	Correlative video-light-electron microscopy demonstrated that the internalized cells were within single-membrane vacuoles.	[12]

## 6. Concluding Remarks

The precise signal that triggers LC3 recruitment to the phagosome remains to be discovered. The roles played by LC3 in mammalian cells have seemingly become more diverse [58] and it remains to be determined whether there are separate pools of LC3 that can be recruited for specific events. Investigation of whether other members of the mammalian LC3 subfamily contribute to LAP pathway may also yield new understanding of the process.

Distinguishing LAP and canonical autophagy at the ultrastructural level using electron microscopy might be facilitated by the application of CLEM [59] to identify specific LC3-labelled compartments. Of more immediate use in studies of mammalian cells infected by bacteria, would be the discovery of a specific marker of LAP that can readily distinguish it from autophagy, or a pharmacological reagent specifically active against LAP.
